# Contribution of Cervical Proprioception, Vision, and Vestibular Feedback on Reducing Dynamic Head–Trunk Orientation Error in the Yaw Direction

**DOI:** 10.3389/fnins.2021.774448

**Published:** 2022-01-24

**Authors:** Rami Mooti, Hangue Park

**Affiliations:** Department of Electrical and Computer Engineering, Texas A&M University, College Station, TX, United States

**Keywords:** cervical proprioception, head–trunk orientation, joint position error, sensory augmentation, sensory integration

## Abstract

The contribution of cervical proprioception, vision, and vestibular feedback to the dynamic head–trunk orientation error in the yaw direction was investigated to further the understanding over the mechanism of coordination among different sensory modalities for dynamic head–trunk orientation. To test the contribution of each sensory modality, individually and together, to dynamic head–trunk orientation, 10 healthy human subjects participated in the extended cervical joint position error test, measuring the ability of repositioning the head back to the reference orientation after 45° yaw rotation of head or trunk. The error between initial and returned angles was measured. The test was repeated under eight different conditions of sensory feedback, with or without each of three sensory modalities. Each subject completed 64 trials (8 per condition) in a random order for fair comparison. No change was found in bias when one of the three modalities was missing, while variance was largest at the lack of dynamic cervical proprioception. When two of the three modalities were missing (i.e., one of the three modalities was present), both bias and variance were minimum at the presence of cervical proprioception. Additionally, both visual and vestibular feedback was redundant (i.e., no further improvement in both bias and variance), if the other one (visual or vestibular feedback) was present with dynamic cervical proprioception. In sum, the experimental results suggest that dynamic cervical proprioception is the most significant sensory modality for reducing the dynamic head–trunk orientation error in the yaw direction.

## Introduction

Our self-awareness of relative orientation between the head and the trunk in both static and dynamic situations is heavily dependent on sensors within joints and muscles in the cervical spine region. While the head directly uses visual and vestibular information to perceive its orientation, the trunk does not inherently have a sensory system detecting its absolute orientation independently from the head. Rather, the orientation of the trunk is perceived relative to the head *via* the cervical region, which is the rotational medium between the head and the trunk. Indeed, the cervical region, along with the muscles surrounding it, is the critical instrument to provide the perception of the head–trunk orientation. The cervical muscles and tendons cross two or more joints and have multiple attachments to different bones. This redundantly interwound structure helps to deliver feedback of the head–trunk orientation accurately by the integration of information from body sensors such as muscle spindles and the Golgi tendon organ (Richmond and Abrahams, 1979; Kamibayashi and Richmond, 1998). The collection of sensory feedback from the cervical area surrounding the neck, regarding length/velocity changes and applied force at muscles and tendons, is called cervical proprioception. Visual feedback also provides information about the trunk orientation relative to the head (i.e., head–trunk orientation), but it is typically limited, as our gaze is typically centered toward our direction of motion rather than our bodies. Additionally, the conversion and matching processes between information derived from vision and proprioception adds an error, which is often called as a visual-proprioceptive matching error (Smeets et al., 2006).

**FIGURE 1:**
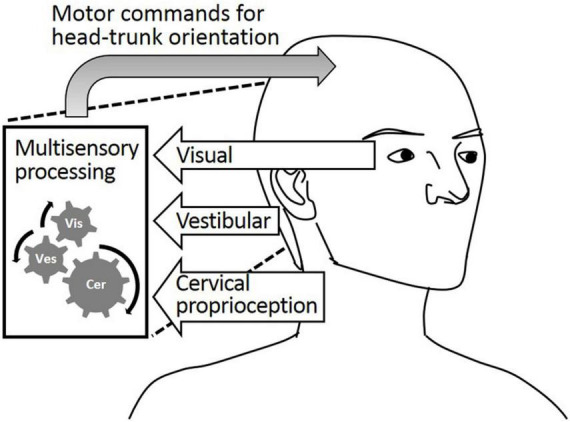
Visual feedback, vestibular feedback, and cervical proprioceptive feedback all major contributors to the head–trunk orientation.

While static head–trunk orientation is mostly perceived by cervical proprioception, the process of perceiving dynamic head–trunk orientation is more complicated. Dynamic head–trunk orientation actively engages head and trunk movements requiring the integration of perceptual feedback from both regions. Dynamic head–trunk orientation is perceived and controlled by the well-orchestrated coordination of cervical proprioception, visual feedback, and vestibular feedback, as described in Figure 1. While proprioception is important, it alone cannot provide holistically accurate perception of the head–trunk orientation under all conditions. The visual and/or vestibular feedback contribute to perceiving the head orientation, which plays an important role in perceiving dynamic head–trunk orientation especially when the trunk not rotated and aid in providing accurate feedback for the relative position of the head and trunk.

Although we know that multiple sensory modalities work in harmony to perceive dynamic head–trunk orientation, as investigated in studies of coordination between cervical proprioception and vestibular feedback (Karnath, 1994; Gdowski and Mccrea, 2000; Luan et al., 2013) and between visual and vestibular feedback (McConville et al., 1994; Karnath et al., 2002), quantification of the contribution of each sensory modality in relation to one another is still missing. This study aims to garner a better understanding of the contribution of each of these the three sensory modalities contributions to the perception of dynamic head–trunk orientation. We are especially interested in the impact of the nervous system’s natural compensation of a limited modality by the intrinsic up-regulation of the remaining sensory modalities.

We first hypothesize that the lack of dynamic cervical proprioception (i.e., cervical proprioception detecting the dynamics of neck rotation) cannot be naturally compensated by visual and vestibular feedback, in dynamic head–trunk orientation. This line of thought derives from the belief that cervical proprioception is the sensory modality the body relies on the most for dynamic head–trunk orientation. Note that proprioception around the cervical area (i.e., cervical proprioception) is specialized for perceiving the head–trunk orientation, while visual and vestibular feedbacks indirectly contribute to the head–trunk orientation *via* an extrinsic reference frames or perception of head rotation itself. We also hypothesize that both visual and vestibular feedback provide information redundant to that provided by cervical proprioception, in dynamic head–trunk orientation. In other words, the dynamic head–trunk orientation error will not be changed by the lack of visual and/or vestibular feedback. Indeed, prior works suggested that up-regulation of cervical proprioception could successfully compensate for the lack of vestibular or visual functions (Schweigart et al., 2002; Nunzio and Schieppati, 2006). However, without an analytical test to measure and provide quantitative data on the contribution of each of these sensory modalities, the capability to analyze and predict this compensatory effect will remain uncertain.

## Materials and Methods

### Data Analysis Using Bias and Variance

**FIGURE 2:**
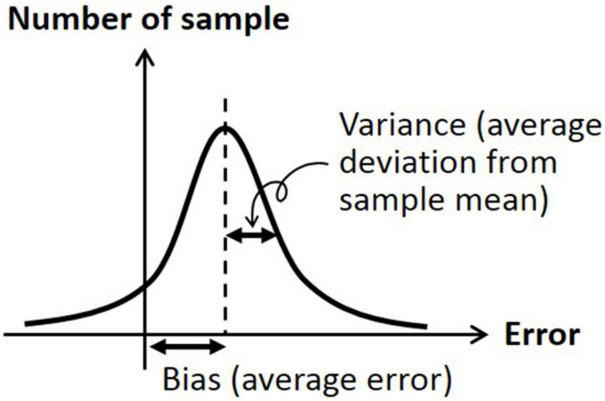
Graphical description of bias (average signed error) and variation (average deviation from sample mean).

The data were analyzed by two metrics; bias was defined as the signed difference between the head orientation angle at the beginning and the head orientation angle at return, and variance was defined as the absolute (unsigned) difference between the average of head orientation angles at return and each of the actual head orientation angle at return (Westgard and Lott, 1981). Accordingly, bias indicates the difference from the target orientation angle (i.e., accuracy measure) and variance indicates the difference from the average of biased orientation angle (i.e., precision measure). See Figure 2 for graphical description of bias and variance.

### Subjects Recruitment and Statistical Test

All experiments were performed adhering to relevant guidelines and regulations, in accordance with the procedure described in the protocol approved on February 28, 2020 by Institutional Review Board, Texas A&M University (IRB2019-1210D). Ten healthy human subjects (five males, five females), aged 20–26 (average 21.9), participated in the study. None of the subjects had a known history of neurological disease, vestibular damage, cervical muscle proprioceptive defects, or visual impairment after correction by lens. All subjects provided their informed consents for the experimentation according to the IRB-approved protocol.

As a statistical analysis, three-way ANOVAs were performed as a full factorial analysis by SPSS (IBM, Chicago, IL, United States) to test effects of the three sensory modalities (vestibular feedback, visual feedback, and dynamic cervical proprioception) and interactions between them, respectively, on the bias and variance. Additionally, multiple one-way ANOVA tests were performed for comparisons between the specific sensory conditions, to determine if the lack of one or two sensory modalities can be naturally compensated by the other remaining sensory modalities. To verify that the data satisfies the prerequisites for the ANOVA test, we tested the normality of the data distribution using the Kolmogorov–Smirnov test of normality. All datasets satisfied the condition of *p* > 0.05 and normality could be assumed. Bonferroni correction was applied for all statistical tests to counteract the problem of multiple comparisons. The significance level was set at *p* = 0.05 (95% confidence interval) for all statistical tests.

### Extended Version of Cervical Joint Position Error Test

The cervical joint position error (JPE) test is a widely accepted method to evaluate the cervical proprioception (Schweigart et al., 2002; Lark and McCarthy, 2007, 2009; Uremović et al., 2007). Although the JPE test is an indirect evaluation of the function of muscle spindles, it is a common practice as the highly concentrated muscle spindles over the cervical area are hard to access and evaluate (Boyd-Clark et al., 2002). In the basic JPE test, subjects were asked to turn their heads from straight-ahead position to a given direction with a certain degree of rotation and then asked to return their heads back to the initial position (straight-ahead position), while blocking the vision. The difference in degrees between where the subjects began the trial and where they ended the trial is used to evaluate the cervical proprioception.

Although the basic JPE test can evaluate the contribution of sensory modalities to dynamic head–trunk orientation while excluding visual feedback, the effect of vestibular and cervical proprioceptive feedback is still combined. Also, the sole effect of visual feedback on dynamic head–trunk orientation cannot be evaluated. Therefore, to evaluate the contribution of visual, vestibular, and cervical proprioceptive feedback, respectively, on head–trunk orientation, we designed an extended version of the JPE test (i.e., extended JPE test).

**FIGURE 3:**
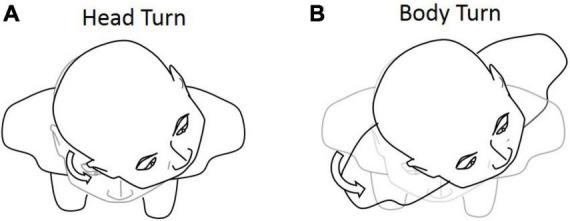
Graphical representation of the **(A)** head-turn (HT) and **(B)** body-turn (BT) tests. Participants were asked to return to the initial position after the 45° rotation.

In the extended JPE test, we first added the body-turn (BT) test in addition to the head-turn (HT) test, to exclude the effect of dynamic cervical proprioception on head–trunk orientation (see Figure 3). In the BT test, subjects were seated in a rotating chair, first facing a “straight-ahead” position, and then passively rotated by the operator to a certain degree. Subjects were asked to keep the head aligned straight forward in regards to the trunk while sitting in an upright position. The chair was then rotated back by the operator, until subjects told the operator to stop. Subjects were asked to say “stop” when they felt the head and body were returned back to the initial orientation.

Additionally, in the extended JPE test, to minimize the effect of vestibular feedback, we also performed all procedures twice with normal and slow rotation speeds. The normal and slow rotation speeds were instructed verbally for HT test, as “rotate at a normal pace” and “rotate slowly to spend at least 30 s for rotation,” respectively, considering the vestibular perception threshold for perceiving horizontal rotation as 2°⋅s^–1^ (Benson et al., 1989; Pinsault and Vuillerme, 2010). The >30-s rotation time, corresponding to <1.5°⋅s^–1^, will provide a condition to minimize vestibular feedback. A normal pace was defined as anything faster than 20 s, corresponding to 2.25°⋅s^–1^. If the participant returned slower than 20 s, they were asked to redo the trial. We intentionally did not fix the time for returning rotation to limit the participants’ ability of using temporal perception as a cue (e.g., memory, counting), as they can subconsciously use the temporal cue saved from the trial done with the best sensory condition.

In sum, the extended JPE test was composed of eight different conditions of sensory modalities (see Table 1) to evaluate the contribution of visual, vestibular, and dynamic cervical proprioceptive feedback, respectively, on dynamic head–trunk orientation. Each condition of the intervention was repeated for 8 times, with a total of 64 trials. As an intervention to dynamic cervical proprioception, two different types of rotation were provided, HT and BT. As a visual intervention, subjects were blindfolded or not. Vestibular intervention was provided by changing the head rotation speed at return, with two different rotation speeds: normal and slow.

**TABLE 1:** Tests with associated sensory modalities involved in each test condition.

No.	Test condition			Sensory modality involved in the condition		
	Turning	Rotation speed	Vision	Cervical proprioception	Visual	Vestibular
1	Head	Normal	Open	✓	✓	✓
2	Head	Slow	Open	✓	✓	
3	Head	Normal	Blocked	✓		✓
4	Head	Slow	Blocked	✓		
5	Head + body	Normal	Open		✓	✓
6	Head + body	Slow	Open		✓	
7	Head + body	Normal	Blocked			✓
8	Head + body	Slow	Blocked			

### Placement of Reflective Markers for Calculating Rotation Angle

**FIGURE 4:**
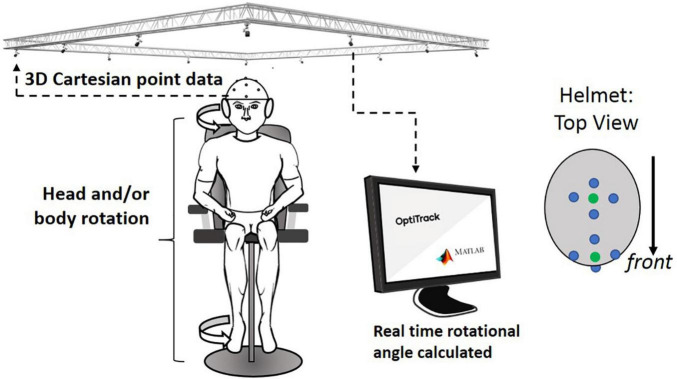
Overall experimental setup: motion capture system received 3D locational information from retro-reflective spheres placed on helmet. This information was sent to a computer that calculated the rotational angle of the helmet from the initial orientation. Four-retro reflective spheres (blue dots in helmet: *top view*) are used to create virtual spheres (green dots that are created in the central location of each of the two sets of the four dots; these green dots were used to indicate the head orientation).

Two sets of four markers, for a total of eight markers, were located on the helmet. The first set of four markers were placed at the front center and another set of four markers were placed around the top center (see Figure 4). In the center of each set, a virtual dot was created, which formed a vector used to calculate the rotation angle. Optical motion capture system (Prime 41, Motive: OptiTrack) provided virtual dots in a 3D Cartesian plane representing real space, by reconstructing actual marker positions within ±0.1 mm of the markers’ real-world locations. A signal processing algorithm in the computer communicated with the Motive software in real time and calculated the head rotation angle. Any initial offset specific to each subject was cancelled at initial calibration at the “straight-ahead” position. The head rotation angle throughout the entire test period was saved with a sampling rate of 16 samples/s.

### Actual Test Procedure

All subjects were asked to sit on the rotational chair placing their feet on the footrest. All subjects were also asked to wear a helmet with eight retro-reflective spherical markers mounted on it (see Figure 4). Subjects were also asked to wear a headphone that projected white noise to reduce the ability to use auditory cues from the surroundings. In the practice trial before the extended JPE test, subjects were guided to rotate their head (HT) by 45° with the verbal stop signal of the operator, repeated twice. The practical trials were used to let the subjects know how much they needed to rotate their head relative to the trunk to reach the head–trunk angle of 45°. Also, note that the all experiments have been conducted in the middle of the laboratory with tables and computers located all around, which can be used as a visual cue in the rotation with visual feedback.

For the HT test, subjects were asked to rotate their head by 45° without guidance, by either normal or slow speed according to the test condition. Subjects were then asked to rotate their head back to the original head–trunk orientation, after completely stopping at the rotated angle. For the BT test, the operator rotated subjects by 45° using the rotational chair. After completely stopping at the rotated angle, the operator rotated subjects back to the original trunk orientation by normal or slow speed, until subjects’ verbal stop signal. For each subject, the extended JPE test with eight different experimental conditions were conducted for 64 times (8 times per each condition) in a random order. Subjects had a rest in the chair between the trials for 1 min. The 64 trials took about 120 min to complete for each subject.

## Results

### Overall Summary of the Full Factorial Three-Way ANOVA Test, Regarding the Effect of Each Sensory Modality and Their Combinations on Bias and Variance

Based on full factorial three-way ANOVA test on bias, both vestibular feedback and dynamic cervical proprioception decreased bias (*p* = 0.002, η^2^ = 0.017 for vestibular feedback and *p* < 0.001, η^2^ = 0.037 for dynamic cervical proprioception), while visual feedback did not change bias (*p* = 0.075, η^2^ = 0.006). The *p*-value for the interaction between vestibular feedback and dynamic cervical proprioception was 0.002 (η^2^ = 0.017), which indicates that vestibular feedback and dynamic cervical proprioception have statistically meaningful interaction on decreasing bias. The *p*-values for other interactions were larger than 0.05.

Based on full factorial three-way ANOVA test on variance, both visual feedback and dynamic cervical proprioception decreased variance (*p* < 0.001, η^2^ = 0.043 for visual feedback and *p* < 0.001, η^2^ = 0.134 for dynamic cervical proprioception), while vestibular feedback did not change variance (*p* = 0.152, η^2^ = 0.004). The *p*-value for the interaction between visual feedback and dynamic cervical proprioception is 0.007 (η^2^ = 0.013), which indicates that visual feedback and dynamic cervical proprioception have statistically meaningful interaction on decreasing variance. The *p*-values for other interactions were larger than 0.05. Table 2 is a summary table of the full factorial three-way ANOVA results.

**TABLE 2:** Full factorial three-way ANOVA test results.

Dependent variable	Factor	Full factorial three-way ANOVA results		
		*F*-value	*p*-Value	Eta-squared (η^2^)
Bias	Vestibular	9.722	0.002	0.017
	Visual	3.174	0.075	0.006
	Dynamic cervical proprioception	21.034	< 0.001	0.037
	Vestibular × visual	0.830	0.363	0.001
	Vestibular × proprioception	9.326	0.002	0.017
	Visual × proprioception	1.134	0.287	0.002
Variance	Vestibular	2.504	0.152	0.004
	Visual	24.655	< 0.001	0.043
	Dynamic cervical proprioception	85.337	< 0.001	0.134
	Vestibular × visual	0.717	0.397	0.001
	Vestibular × proprioception	0.114	0.736	< 0.001
	Visual × proprioception	7.459	0.007	0.013

### Effect of Each Sensory Modality on Bias and Variance When One of the Three Sensory Modalities Was Missing

First, we evaluated bias and variance in returning to the straight-ahead position, when one of the three sensory modalities was missing or minimized (comparison among the conditions of 1, 2, 3, and 5). These comparisons will test the first hypothesis that lack of dynamic cervical proprioception cannot be naturally compensated by visual and vestibular feedback in dynamic head–trunk orientation. In the control condition (condition 1; with presence of all sensory modalities), the bias relative to the origin was 0.17° ± 0.22° and the variance was 1.36° ± 0.12° (values are represented as average ± standard error). Note that positive and negative biases indicate overshoot and undershoot in return, respectively. When vestibular feedback was minimized (condition 2), the bias was −0.07° ± 0.24° and the variance was 1.57° ± 0.15°. When visual feedback was missing (condition 3), the bias was 0.14° ± 0.29° and the variance was 1.71° ± 0.18°. When dynamic cervical proprioception was missing (condition 5), the bias was 0.44° ± 0.47° and the variance was 2.93° ± 0.25°.

**FIGURE 5:**
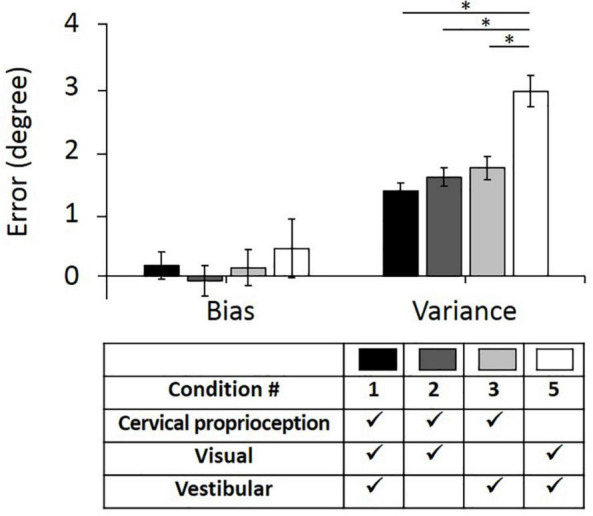
Bias (*left*) and variance (*right*) in head–trunk orientation, with all of three sensory modalities or at the loss of one of the three major sensory modalities for perceiving head–trunk orientation. Error bar indicates the standard error across 80 trials (8 trials per subject) for each condition and asterisk (*) indicates statistical difference with 95% confidence interval.

Bias and variance with all four conditions of sensory modalities and results of comparison between them are summarized in Figure 5. Subjects did not show difference in bias relative to the origin even though one of the three sensory modalities was missing (*p* = 0.465, η^2^ = 0.003 for missing vestibular; *p* = 0.931, η^2^ < 0.001 for missing visual; *p* = 0.599, η^2^ = 0.002 for missing dynamic cervical proprioception). In terms of the variance, there was no statistically significant change when vestibular feedback was minimized (*p* = 0.268, η^2^ = 0.008) or vision was blocked (*p* = 0.105, η^2^ = 0.017). However, variance was increased when dynamic cervical proprioception was missing (*p* < 0.001, η^2^ = 0.171). The variance when dynamic cervical proprioception was missing was larger than the variance when vestibular feedback was minimized (*p* < 0.001, η^2^ = 0.124) and also larger than the variance when vision was blocked (*p* < 0.001, η^2^ = 0.090).

### Effect of Each Sensory Modality on Bias and Variance When Two of the Three Sensory Modalities Were Missing

Second, we evaluated the bias and variance when two of the three major sensory modalities were missing (i.e., only one of the three major sensory modalities was provided for perceiving head–trunk orientation). These comparisons were performed to provide the idea of how important each sensory modality is in the absence of other sensory modalities. We used condition 8 as a control condition, where all three sensory modalities were missing or minimized. In the control condition (condition 8), the bias was 5.06° ± 2.05° and the variance was 11.62° ± 1.04°. Three conditions, where only one of the three sensory modalities was present, were then compared with the condition 8 and with each other. When only vestibular feedback was present (condition 7), the bias decreased to 1.37° ± 0.80° and the variance decreased to 4.74° ± 0.47°. When only visual feedback was present (condition 6), the bias decreased to 2.97° ± 0.47° and the variance decreased to 2.97° ± 0.28°. When only dynamic cervical proprioception was present (condition 4), the bias decreased to 0.72° ± 0.42° and the variance decreased to 2.38° ± 0.26°.

**FIGURE 6:**
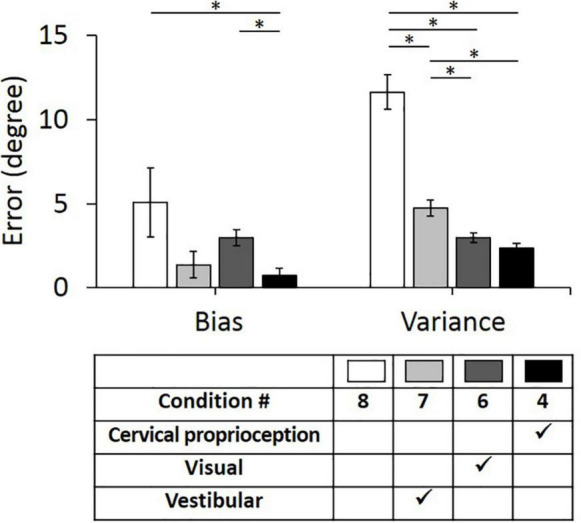
Bias (*left*) and variance (*right*) in head–trunk orientation, with none of three sensory modalities or at the presence of one of the three major sensory modalities for perceiving head–trunk orientation. Error bar indicates the standard error across 80 trials (8 trials per subject) for each condition and asterisk (*) indicates statistical difference with 95% confidence interval.

The bias and variance with all four conditions of sensory modalities and results of comparison between them are summarized in Figure 6. While the presence of dynamic cervical proprioception reduced the bias (*p* = 0.040, η^2^ = 0.027), compared to the case with none of three sensory modalities, the presence of vestibular feedback or visual feedback did not reduce the bias (*p* = 0.096, η^2^ = 0.017 for vestibular feedback; *p* = 0.322, η^2^ = 0.006 for visual feedback) compared to the case with none of three sensory modalities. Also, the bias with only dynamic cervical proprioception is smaller than the bias with only visual feedback (*p* < 0.001, η^2^ = 0.075), while it is not different from the bias with only vestibular feedback (*p* = 0.473, η^2^ = 0.003). In terms of the variance, subjects did perform more precisely when any one of three sensory modalities was present (*p* < 0.001, η^2^ = 0.189 for vestibular; *p* < 0.001, η^2^ = 0.292 for visual; and *p* < 0.001, η^2^ = 0.322 for dynamic cervical proprioception). Also, subjects did perform the most precisely (i.e., minimum variance) when only dynamic cervical proprioception or only visual feedback was present. The variance with only dynamic cervical proprioception was smaller than the variance with only vestibular feedback (*p* < 0.001, η^2^ = 0.110), and the variance with only visual feedback was smaller than the variance with only vestibular feedback (*p* = 0.001, η^2^ = 0.063). The variance was not different between the cases of only visual feedback and of only dynamic cervical proprioception (*p* = 0.124, η^2^ = 0.015).

### Effect of Vestibular and Visual Feedback on Bias and Variance, on Top of Dynamic Cervical Proprioception

**FIGURE 7:**
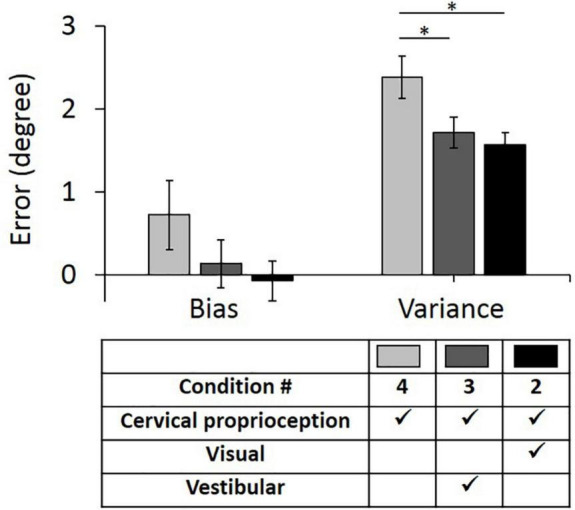
Bias (*left*) and variance (*right*) in head–trunk orientation, at the presence of dynamic cervical proprioception with or without visual and/or vestibular feedback. It is to investigate the redundancy of visual and/or vestibular feedback at the presence of dynamic cervical proprioception. Error bar indicates the standard error across 80 trials (8 trials per subject) for each condition and asterisk (*) indicates statistical difference with 95% confidence interval.

The results of the full factorial three-way ANOVA test suggest that vestibular feedback plays an important role in decreasing bias and visual feedback plays an important role in decreasing variance. Further, the results suggest that vestibular feedback and dynamic cervical proprioception have statistically meaningful interaction on decreasing bias and visual feedback and dynamic cervical proprioception have statistically meaningful interaction on decreasing variance. To test the second hypothesis that vestibular and visual feedback provide information redundant to dynamic cervical proprioception in dynamic head–trunk orientation, we conducted two additional one-way ANOVA tests between condition 4 (only dynamic cervical proprioception) and condition 3 (dynamic cervical proprioception + vestibular feedback), and between condition 4 and condition 2 (dynamic cervical proprioception + visual feedback). Note that we used condition 4 as a control condition, where only dynamic cervical proprioception was present. In the control condition (condition 4), the bias was 0.72° ± 0.42° and the variance was 2.34° ± 0.26°. Two conditions, where either vestibular or visual feedback was present together with dynamic cervical proprioception, were then compared with the condition 4 and with each other. When vestibular feedback was present together with dynamic cervical proprioception (condition 3), the bias was 0.14° ± 0.29° and the variance was 1.71° ± 0.18°. When visual feedback was present together with dynamic cervical proprioception (condition 2), the bias was −0.07° ± 0.24° and the variance was 1.57° ± 0.15°. The bias and variance with all three conditions of sensory modalities and results of comparison between them are shown in Figure 7. Bias was not decreased by the addition of either vestibular or visual feedback (*p* = 0.248, η^2^ = 0.008 for vestibular feedback; *p* = 0.101, η^2^ = 0.017 for visual feedback), while variance was decreased by the addition of either vestibular or visual feedback (*p* = 0.036, η^2^ = 0.027 for vestibular feedback; *p* = 0.007, η^2^ = 0.045 for visual feedback).

## Discussion

### Lack of Dynamic Cervical Proprioception Can Be Compensated by the Other Two Sensory Modalities Together in Keeping Accuracy (Bias) of Dynamic Head–Trunk Orientation, but Not in Keeping Precision (Variance)

Previous studies suggest that increased neck orientation error, caused by damage or fatigue in the cervical muscles, could not be compensated by intact visual and vestibular feedback (Pinsault et al., 2010). This is expected as defects in dynamic cervical proprioception have shown to cause significant deficiency in body control, motor output, and perceived body orientation (Quoniam et al., 1995; Koskimies et al., 1997; Gosselin et al., 2004; Madeleine et al., 2004; Stapley et al., 2006; Kogler et al., 2009; Bianco et al., 2014; Malmström et al., 2017). Redundantly interwound structures of muscles/tendons and high density of muscle spindles in the cervical region suggest this region has a significant contribution toward dynamic cervical proprioception in perceiving dynamic head–trunk orientation (Boyd-Clark et al., 2002). Our experimental results partially agree with and partially contradict with this prior knowledge, showing vestibular and visual feedback together successfully compensated for the lack of dynamic cervical proprioception in keeping accuracy (bias) of dynamic head–trunk orientation but not in keeping precision (variance).

Our experimental results showed that the lack of dynamic cervical proprioception significantly increased the variance (by ∼116%), even while the other two sensory modalities (vestibular and visual feedback) were still present. The lack of either vestibular or visual feedback did not change the variance, given that one of these is present with dynamic cervical proprioception. This result suggests that any defect in dynamic cervical proprioception could not be compensated for by the other two sensory modalities in keeping the precision of dynamic head–trunk orientation, while the lack of vestibular or visual feedback could be compensated by the remaining two sensory modalities. This result agrees with the prior reports that defect of dynamic cervical proprioception cannot be compensated for by intact visual and vestibular feedback. These results also support our first hypothesis that the lack of dynamic cervical proprioception cannot be naturally compensated for by visual and vestibular feedback in dynamic head–trunk orientation.

However, in terms of accuracy (bias), our experimental results showed that the lack of any single sensory modality did not change the bias if the other two sensory modalities are present. This result suggests that the defect of any single sensory modality (even in dynamic cervical proprioception) could be compensated by the two other sensory modalities, in keeping the accuracy of dynamic head–trunk orientation. We interpret this discrepancy between prior works and this works as follows: Having defects in dynamic cervical proprioception is more problematic in our experimental setting than a lack of dynamic cervical proprioception. In other words, having an erroneous signal may disrupt the perception worse than having no signal.

### Lack of Either Vestibular or Visual Feedback Can Be Compensated by Dynamic Cervical Proprioception in Keeping Accuracy (Bias) of Dynamic Head–Trunk Orientation, but Not in Keeping Precision (Variance)

Results of previous studies suggest that bilateral vestibular loss has minimal effect on head–trunk orientation (Malmström et al., 2009). However, it is not clear if this compensation is the result of up-regulation of cervical proprioception or natural compensation (i.e., redundancy of vestibular feedback on top of cervical proprioception). Additionally, the redundancy of visual feedback on top of cervical proprioception is also not known. We therefore tested whether vestibular or visual feedback provides redundant information already provided from cervical proprioception in perceiving dynamic head–trunk orientation, with an experimental setup designed to identify the effect of each sensory modality individually and their combinations.

Our experimental results showed that variance decreased with the addition of either vestibular or visual feedback on top of dynamic cervical proprioception, while bias did not change with the addition of either vestibular or visual feedback on top of dynamic cervical proprioception. This result suggests that vestibular and visual feedback is not redundant for dynamic cervical proprioception and add value for keeping the precision of dynamic head–trunk orientation. These results reject our second hypothesis that both vestibular and visual feedback provide information redundant to dynamic cervical proprioception in dynamic head–trunk orientation.

However, both vestibular and visual feedback was redundant when the other modality was present with dynamic cervical proprioception. Neither minimization of vestibular feedback nor lack of visual feedback changed variance as well as bias, in instances where dynamic cervical proprioception was present with either vestibular or visual feedback. This result suggests that both vestibular and visual feedback become redundant in both accuracy and precision of dynamic head–trunk orientation, if dynamic cervical proprioception was already present with either vestibular or visual feedback alone.

### Vestibular Feedback Provides More Significant Contribution Than Visual Feedback in Keeping Accuracy (Bias) of Dynamic Head–Trunk Orientation, and Vice Versa in Keeping Precision (Variance)

Previous experimental results suggested that reliance on visual feedback created a higher bias in head orientation error, compared to the case of relying on vestibular feedback (Crane, 2012), suggesting that vestibular feedback plays a more important role than visual feedback in keeping accuracy. Additionally, in natural instances of aging, the body has been found to increase reliance on visual feedback to compensate for the motor loss at tasks that required high precision (Coats and Wann, 2011), suggesting that visual feedback plays an important role in keeping precision. The presented work supports both of these prior findings. Our three-way ANOVA test results suggest that vestibular feedback decreased bias while visual feedback did not change bias. Furthermore, visual feedback decreased variance while vestibular feedback did not change variance. Therefore, we confirmed the previous experimental results that vestibular feedback is more effective than visual feedback in reducing the bias in head–trunk orientation (i.e., enhance accuracy), while visual feedback is more effective than vestibular feedback in reducing the variation (i.e., enhance precision and reproducibility) in dynamic head–trunk orientation.

The different effects between vestibular and visual feedback can also be explained by the different characteristics of information provided by each sensory modality. For horizontal head rotation in the yaw direction, like the given experimental setup, vestibular feedback provides information regarding head rotation by intrinsic reference frame, while visual feedback provides spatial information of the head relative to external objects (i.e., extrinsic reference frame). Using an extrinsic reference frames is perhaps advantageous for precision, as external objects can be used by humans to consistently arrive at a certain location. However, any bias caused by external objects may be hard to be addressed, without the other sensory modalities.

### Defects in Dynamic Cervical Proprioception Might Be Better Addressed by Direct Cervical Proprioceptive Augmentation Than Indirect Visual or Vestibular Augmentation

Dynamic cervical proprioception can become dysfunctional, which results in serious balance problems (Childs et al., 2008; Field et al., 2008). Experimental results suggest that the combination of vestibular and visual feedback could not compensate for the lack of dynamic cervical proprioception, in terms of the variance. In other words, intrinsic up-regulation of vestibular and visual feedback was not enough to compensate for the lack of dynamic cervical proprioception. In this regard, direct proprioceptive modulation (or proprioceptive illusion) on the cervical region might better address the problem in dynamic cervical proprioception, instead of indirect visual or vestibular augmentation. Multiple approaches to proprioceptive modulation have shown promising results although there is a long way to secure the consistency of evoked perception (Bove et al., 2002; Hellström et al., 2005; Petterossi and Schieppati, 2014; Azbell et al., 2020; Zhao et al., 2020; Rangwani and Park, 2021). Increasing the consistency/robustness of the intervention while also integrating a solution to be wearable and accessible in daily lives are remaining tasks for the direct augmentation of cervical proprioception.

### Limitation of This Work

This work has limitation in multiple respects. First, we only tested the hypotheses with healthy human subjects having no defect in any of three sensory modalities (i.e., vision, vestibular, and cervical proprioception). Although we tested dynamic head–trunk orientation at conditions when part of three major sensory modalities is missing or minimized, the neural mechanism of the emulated conditions should be further investigated to apply the findings to cases of the actual defective sensory conditions. Second, the number of human subjects was small (10) and the age range was small (20–26 years), which should be increased in the next experiment for more reliable conclusion over biological variation. Third, the efficacy of the extended JPE test needs to be further demonstrated with follow-up experiments, to confirm fair comparison between the BT and HT conditions. For example, differences between the BT and the HT conditions may be affected by overall body posture and the BT condition may increase the cognitive load required to accomplish the task, compared to the HT condition. Lastly, we didn’t separately consider the proprioception from the eye muscle changed by gaze direction, even though multiple prior works reported its significance in dynamic head–trunk orientation (Cohen, 1961; Roll et al., 1991). Although we expect the gaze movement and the resulting proprioception from the eye muscle would have been minimized in our setup with blindfolded participants, it would be worthwhile to test the effect of the gaze movement separately in subsequent works.

## Conclusion

We investigated the contribution of dynamic cervical proprioception, vision, and vestibular feedback toward reducing bias (accuracy measure) or variance (precision measure) in dynamic head–trunk orientation across the horizontal plane in yaw direction. To evaluate the individual effect of dynamic cervical proprioception, vision, and vestibular feedback during dynamic head–trunk orientation, we introduced a BT test (lack of dynamic cervical proprioception) and slow rotation (vestibular minimization) to the classical cervical JPE test (i.e., extended JPE test). Experimental results suggest that, in general, dynamic cervical proprioception contributed more significantly than the other two sensory modalities (vestibular and visual feedback) to reducing both bias and variance in dynamic head–trunk orientation. In cases of lacking one or two of these three major sensory modalities, dynamic cervical proprioception showed the most significant impact. This work provides a contribution map of each sensory modality on reducing the error in the dynamic yaw head–trunk orientation, in terms of both accuracy and precision (i.e., reducing bias and variance). These results will enhance the accuracy of diagnosis and help people design the best sensory augmentation approach for a given condition of deficiency of sensory modalities for perceiving dynamic head–trunk orientation.

## Data Availability Statement

The raw data supporting the conclusions of this article will be made available by the authors, without undue reservation.

## Ethics Statement

The studies involving human participants were reviewed and approved by the Institutional Review Board of Texas A&M University. The patients/participants provided their written informed consent to participate in this study.

## Author Contributions

HP and RM conceptualized the idea and designed the experiment, got the experimental protocol approved for human subject experiments, analyzed the experimental data, and wrote the manuscript. RM prepared the electrical system for experiments, recruited the human subjects, and collected the experimental data. Both authors contributed to the article and approved the submitted version.

## Conflict of Interest

The authors declare that the research was conducted in the absence of any commercial or financial relationships that could be construed as a potential conflict of interest.

## Publisher’s Note

All claims expressed in this article are solely those of the authors and do not necessarily represent those of their affiliated organizations, or those of the publisher, the editors and the reviewers. Any product that may be evaluated in this article, or claim that may be made by its manufacturer, is not guaranteed or endorsed by the publisher.
